# Auto-Calibration and Fault Detection and Isolation of Skewed Redundant Accelerometers in Measurement While Drilling Systems

**DOI:** 10.3390/s18030702

**Published:** 2018-02-27

**Authors:** Seyed Mohsen Seyed Moosavi, Bijan Moaveni, Behzad Moshiri, Mohammad Reza Arvan

**Affiliations:** 1 Department of Electrical Engineering, Science and Research Branch, Islamic Azad University, Tehran 14778-9385, Iran; m.moosavi@srbiau.ac.ir; 2 School of Railway Engineering, Iran University of Science and Technology, Tehran 16846-13114, Iran; 3 Control and Intelligent Processing, Center of Excellence, School of Electrical and Computer Engineering, University of Tehran, Tehran 14395-515, Iran; moshiri@ut.ac.ir; 4 Department of Electrical Engineering, Malek-Ashtar University of Technology, Tehran 15875-1774, Iran; arvan@mut.ac.ir

**Keywords:** accelerometer, auto-calibration, directional drilling, fault diagnosis and isolation, measurement while drilling (MWD), optimal skewed structure

## Abstract

The present study designed skewed redundant accelerometers for a Measurement While Drilling (MWD) tool and executed auto-calibration, fault diagnosis and isolation of accelerometers in this tool. The optimal structure includes four accelerometers was selected and designed precisely in accordance with the physical shape of the existing MWD tool. A new four-accelerometer structure was designed, implemented and installed on the current system, replacing the conventional orthogonal structure. Auto-calibration operation of skewed redundant accelerometers and all combinations of three accelerometers have been done. Consequently, biases, scale factors, and misalignment factors of accelerometers have been successfully estimated. By defecting the sensors in the new optimal skewed redundant structure, the fault was detected using the proposed FDI method and the faulty sensor was diagnosed and isolated. The results indicate that the system can continue to operate with at least three correct sensors.

## 1. Introduction

Directional drilling refers to the type of drilling where the upper part of the reservoir cannot be accessed on land due to buildings or gravity. Drilling starts from adjacent areas and after a certain amount of drilling, starts on a detour and continues to the desired point using an internal tubing cutter [1]. In achieving this predetermined goal, a number of parameters need to be measured using various types of Measuring While Drilling (MWD) tools. However, one of the major problems of directional drilling for oil and gas is the loss of bit positions due to MWD errors or failures [2]. According to field surveys in drilling operations, these problems are caused by initial errors of the sensors due to manufacturer error or improper sensor installation. This problem will have a negative impact during the drilling operations, such as trying to free the drill pipes, heat, inner layers of the earth and the occurrence of magnetic materials, sequential activation of sensors to save energy, and vibration on the drill strings. Consequently, the sensor parameters in accelerometers, such as scale factor and sensor bias, often change [2]. This will cause the sensors performance error and change the calibration coefficients. In addition, it will cause faults and may even destroy the sensors.

Lack of redundant sensors in the system and not performing auto-calibration in drilling operation affect the faults or errors of the accelerometers, therefore, drilling operation must be stopped. In this case, the drilling device must be removed from the well and a newly calibrated MWD should be immediately inserted into the well. This operation will stop the production process, causing loss of the well, and increase production costs. Therefore, the purpose of this study is to design skewed redundant accelerometers as a drilling measurement system that performs auto-calibration process and fault diagnosis, and isolation of faulty accelerometers. The use of redundant sensors will improve the reliability and accuracy of the navigation system. 

An important point about redundant sensors is determining its optimal structure and in this case a number of studies have been conducted [3,4,5,6,7,8]. They used figures of merit (FOMs) to evaluate the position sensor structure and proper placement for navigation function as well as fault detection and isolation (FDI). For example, Jia [7] analyzed various methodologies for combining the data in the Air Navigation System by considering FDI performance. Shim et al. [8] proposed a method for providing the optimal sensor structure from both perspectives of navigation and FDI performances. They showed that the symmetrical structures, such as Platonic bodies, are the best structures for both FDI and navigation performance.

In this study, a 4-sensor structure based on the optimal structures proposed by Shim et al. [8] has been selected and designed using an existing MWD tool. In the proposed system, the new four accelerometer structure is operationally built, installed, and used replacing a three orthogonal accelerometer system. 

For auto-calibration of three orthogonal accelerometers during drilling operation, a general approach such as the use of gravitational acceleration vector for the Earth and combination of the problem with nonlinear optimization methods can be applied [9,10,11,12,13,14,15,16,17]. The main idea is that the orthogonal accelerometer structure is considered in the majority of existing studies.

Li and Duan [11] used the Lyunberg-Marquardt algorithm based on a linearization analysis. Frosio et al. [12,16] executed auto-calibration using data from the sensor datasheet and applied Taylor's first or second order approximation and related analysis for optimization and application of Newton's algorithm. Brage et al. [13] performed auto-calibration for wearable accelerometers using the Moving Average Value method. In the work of Ye and Su [15], a nonlinear function of the sum of squared errors was linearized and the minMax function was used for optimization. These methods are sensitive to the initial data and lead to divergence if the data is not appropriate. Frosio et al. [17] used the maximum likelihood estimation for auto-calibration by selecting a system model under certain conditions. 

In this study, auto-calibration of the skewed non-orthogonal redundant accelerometers is performed using the Newton-Raphson non-linear optimization method. Since redundant sensors are used in the proposed MWD, it is crucial to solve the problem of fault diagnosis and isolation.

Various FDI methods can be categorized from different perspectives. They can be categorized into two main groups: data-based methods and model-based approaches. The majority of data-based techniques are categorized according to the comparison between measured values by sensors, the comparison between the predetermined values and the values measured by the sensors based on the desired characteristics and the logical inference [18,19,20,21,22,23]. The data-based algorithms based on features are generally effective. Because these attributes will result in greater resolution in various error situations. Lei et al. [21] suggested some common features of the time signal and frequency domains. Youssef et al. [22] introduced a Proportional Integral observer to estimate the faults in actuators and sensors using the Takagi–Sugeno fuzzy model. Ding et al. [23] presented a data-driven framework for designing an observer-based fault detection. 

On the other hand, there are a number of real physical systems among the dynamic systems that are near to the equilibrium point in a continuous-time operation. The performance of these systems can be modeled by differential or difference equations. Thus, the faults can be diagnosed using a mathematical model and model-based methods [24,25]. In this study, an algorithm for FDI according to the data-based method with the desired feature is proposed. 

The structure of this paper is organized as follows: Section 2 presents the proposed skewed redundant accelerometers (SRAMWD) model. Section 3 describes auto-calibration of SRAMWD. Section 4 provides an algorithm for FDI of the faulty accelerometers in the SRAMWD structure. Section 5 presents the results of the experiments. Section 6 concludes the study following with the contributions of the study and recommendations for future works.

## 2. System Model

In the previous MWD studies, triangular orthogonal accelerometer structures have been investigated. The structure of the skewed non-orthogonal redundant accelerometer system has been studied in the present paper. Assume that n accelerometers with skewed structure are used in the SRAMWD system, the full bias and full scale factor of the *i^th^* sensor are obtained from the following formulas:

(1) $$SF_{i}^{*}=SF_{i}+SFT1_{i}{\left(T_{S}-T_{C}\right)}^{1}+SFT2_{i}{\left(T_{S}-T_{C}\right)}^{2}+SFT3_{i}{\left(T_{S}-T_{C}\right)}^{3}$$

(2) $$B_{i}^{*}=B_{i}+BT1_{i}{\left(T_{S}-T_{C}\right)}^{1}+BT2_{i}{\left(T_{S}-T_{C}\right)}^{2}+BT3_{i}{\left(T_{S}-T_{C}\right)}^{3}$$

The parameters of Equations (1) and (2) are defined in Table 1. The output of each sensor is the voltage and the acceleration value measured in each direction by the sensors can be calculated based on the following equations:

(3) $$G_{i}=\left(\frac{V_{i}}{SF_{i}^{*}}-B_{i}^{*}\right)-\overset{n}{\underset{k=1}{\sum }}m_{ik}\left(\frac{V_{k}}{SF_{k}^{*}}-B_{k}^{*}\right)$$

The measurement equation for redundant accelerometers is presented as follows:

(4) $$\left[\begin{array}{c} G_{1}\\ \begin{array}{c} G_{2}\\ \vdots \\ G_{n} \end{array} \end{array}\right]=H\cdot \left[\begin{array}{c} G_{X}\\ G_{Y}\\ G_{Z} \end{array}\right]+\varepsilon \left(t\right)\;,\;\varepsilon \sim N\left(0,\rho I_{n}\right)$$

in which: $G_{n\times 1}={\left[G_{1}G_{2}\ldots G_{n}\right]}^{T}$ is the acceleration measured by the accelerometers in line with it. $H_{n\times 3}={\left[h_{1}\ldots h_{n}\right]}^{T}$ is the measuring matrix with rank(H)=3 and $\left|h_{i}\right|=1\;,\;i=1,2,\ldots ,n$ and $\varepsilon \left(t\right)={\left[\varepsilon_{1}\varepsilon_{2}\ldots \varepsilon_{n}\right]}^{T}\in R^{n}\;$ is a vector including the measurement zero mean white noises with a normal distribution.

The triple response of acceleration vector can be calculated using the least squares method, given as follows:

(5) $$\left[\begin{array}{c} G_{X}\\ G_{Y}\\ G_{Z} \end{array}\right]={\left(H^{T}H\right)}^{-1}H^{T}\left[\begin{array}{c} G_{1}\\ \begin{array}{c} G_{2}\\ \vdots \\ G_{n} \end{array} \end{array}\right]$$

The measured gravitational acceleration of the Earth is calculated as follows:

(6) $${\hat{G}}_{L}=\sqrt{G_{X}^{2}+G_{Y}^{2}+G_{Z}^{2}}$$

In this system, calibration of accelerometers is used to estimate the numerical values of the bias parameters, scale factors, and the misalignment parameters of accelerometers. 

## 3. Auto-Calibration of the Accelerometers

In MWD systems, the Earth’s gravitational acceleration is the criterion for auto-calibration of the accelerometers. The error of calculating the Earth’s gravitational acceleration is the difference between $G_{L}$, the actual value of Earth’s gravitational acceleration, and the amount of acceleration resulting from the accelerometer measurement. The error in each sample is calculated as follows:

(7) $$e_{g}\left(k\right)={{\hat{G}}_{L}}^{2}\left(k\right)-{G_{L}}^{2}=G_{X}^{2}\left(k\right)+G_{Y}^{2}\left(k\right)+G_{Z}^{2}\left(k\right)-{G_{L}}^{2}$$

An approximate value for Earth’s gravitational acceleration at a given latitude can be calculated from the following formula [26]:

(8) $$G=9.780327\left(1+A\sin^{2}\left(L\right)-B\sin^{2}\left(2L\right)\right)$$

where: A=0.0053024; B=0.0000058; *L* = latitude. The formula for determining the gravitational acceleration in depth of the Earth is:

(9) $$G_{L}=\frac{R_{e}-d}{R_{e}}G$$

where: $R_{e}=\mathrm{radius}\;\mathrm{of}\;\mathrm{the}\;\mathrm{Earth}$; *d* = the depth in meters of the point inside the Earth. 

The instrument is initially sampled at *N* different positions and the temperature and output voltage of the accelerometers are measured. The total normalized squared error of gravitational acceleration measurement at *N* different positions is obtained using the following equation:

(10) $$E_{g}=\frac{\sum_{k=1}^{N}e_{g}^{2}\left(k\right)}{N}$$

where $E_{g}$ is a nonlinear function of the sensor parameters. The parameters are measured based on observed data using nonlinear optimal minimization methods such as the nonlinear least squares method. For initial values of the parameters, the parameters supplied by the manufacturer are used for each type of installed sensor structure. Based on the Newton-Raphson method, the following formula is often used:

(11) $$\theta^{n+1}=\theta^{n}-\alpha \cdot H^{-1}\left(\theta^{n}\right)\cdot J\left(\theta^{n}\right)$$

where $\theta^{n}$ is a vector of unknown parameters (bias, scale factor, and misalignment factors) at the *n*th stage. $J\left(\theta^{n}\right)$ and $H\left(\theta^{n}\right)$ are the Jacobin vector and the Hessian matrix of the error $E_{g}$ respectively. α is the damping parameter. When the following convergence condition is satisfied, the repetition is terminated:

(12) $$\max{\left\{\left|\frac{\theta_{k}^{n}-\theta_{k}^{n-1}}{\left(\theta_{k}^{n}+\theta_{k}^{n-1}\right)/2}\right|\right\}}_{k=1,2,\ldots }<\varepsilon$$

where $\theta_{k}^{n}$ denotes the kth element of the vector θ in the *n*th repetition. ε is a threshold that is experimentally assumed to be $1.5\times 10^{-6}$.

## 4. FDI and Optimal SRAMWD Structure

Shim et al. [8] presented a method for providing an optimal sensor structure in terms of both navigation and FDI performances. Due to the number of sensors, there is a large number of optimal structures from the navigation performance perspective. Among these optimal navigation structures, it is necessary to select the structure that has the best FDI performance. It has been proved that the optimal structure among the structures providing the best navigation performance is the structure that maximizes the angle between the two nearest sensors. Therefore, symmetrical structures such as the Platonic bodies are known as the best structures for both performances of FDI and navigation. 

Given the structure of the skewed redundant accelerometers using at least three redundant accelerometers and writing related equations, it is possible to calculate the G vector and the Earth’s gravitational acceleration based on three accelerometers. Therefore, if a fault occurs in a sensor, by having at least three correct sensors, the defective sensor can be diagnosed by calculating various combinations of sensors and by taking several samples of accelerometers data using proposed statistical characteristic and data-based diagnosis method. 

If there are n sensors in the system, the number of possible combinations of three sensors of the n sensor is as follows:

(13) $$\left(\begin{array}{c} n\\ 3 \end{array}\right)=\frac{n!}{3!\left(n-3\right)!}$$

If *N* sample data are available, the gravitational acceleration should be calculated for all possible combinations in these samples and the average of calculated accelerations errors should be determined. By having an acceptable limit for error, only in the case of using correct sensors, the average of calculated acceleration error is at an acceptable error limit. Therefore, the faulty sensor or sensors can be detected. By analyzing the conventional features extracted from signal in time-domain, the proper feature of the problem is considered based on the Mean Absolute Error (MAE) for the *N* samples of the calculated acceleration average:

(14) $$\mathrm{MAE}=\frac{\sum_{k=1}^{N}\left|\hat{G_{L}}\left(k\right)-G_{L}\right|}{N}$$

The condition of the correctness of the sensors is that the MAE is not greater than an acceptable error limit $\delta_{F}$. If this condition is not met, there is a fault on the sensors.

(15) $$E_{mean}<\delta_{F}$$

**Figure 1:**
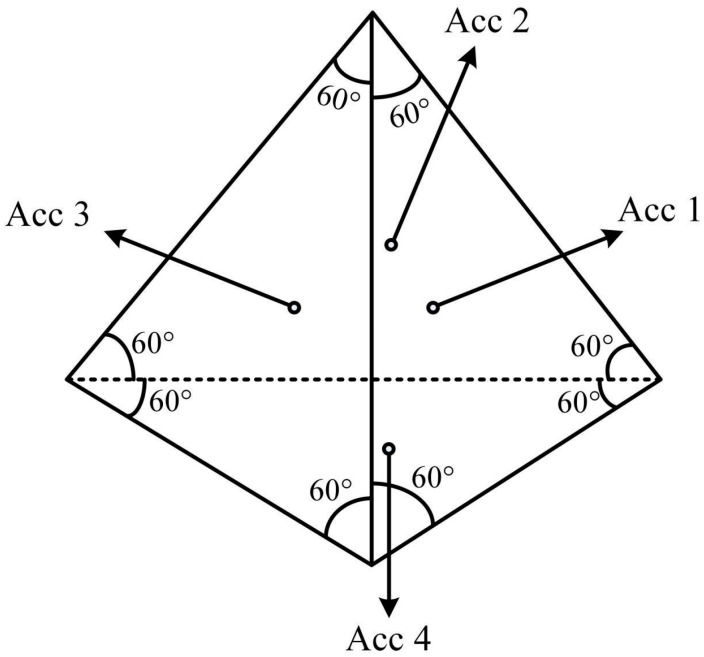
The selected optimal skewed structure of 4-sensor (adapted from Shim and Yang [8]).

In this study, the optimal structure of four redundant sensors, as shown in Figure 1, is considered for the design and implementation of the new system. 

The measurement matrix for this structure is:

(16) $$H=\left[\begin{array}{ccc} \frac{2\sqrt{2}}{3} & 0 & \frac{1}{3}\\ -\frac{\sqrt{2}}{3} & \frac{\sqrt{6}}{3} & \frac{1}{3}\\ -\frac{\sqrt{2}}{3} & -\frac{\sqrt{6}}{3} & \frac{1}{3}\\ 0 & 0 & 1 \end{array}\right]$$

By using all accelerometers in three directions, the results are presented as follows:

(17) $$\left[\begin{array}{c} G_{X}\\ G_{Y}\\ G_{Z} \end{array}\right]={\left(H^{T}H\right)}^{-1}H^{T}\left[\begin{array}{c} G_{1}\\ \begin{array}{c} G_{2}\\ G_{3}\\ G_{4} \end{array} \end{array}\right]=\left[\begin{array}{cccc} \frac{\sqrt{2}}{2} & -\frac{\sqrt{2}}{4} & -\frac{\sqrt{2}}{4} & 0\\ 0 & \frac{\sqrt{6}}{4} & -\frac{\sqrt{6}}{4} & 0\\ \frac{1}{4} & \frac{1}{4} & \frac{1}{4} & \frac{3}{4} \end{array}\right]\left[\begin{array}{c} G_{1}\\ \begin{array}{c} G_{2}\\ G_{3}\\ G_{4} \end{array} \end{array}\right]$$

Therefore, for each data sample, the Earth’s gravitational acceleration is calculated using all the sensors according to Equation (6). There are four possible combinations of three sensors. In the case of combination of accelerometers 2, 3 and 4, the acceleration in different directions can be calculated as follows:

(18) $$\left[\begin{array}{c} G_{X1}\\ G_{Y1}\\ G_{Z1} \end{array}\right]=\left[\begin{array}{ccc} -\frac{3\sqrt{2}}{4} & -\frac{3\sqrt{2}}{4} & \frac{\sqrt{2}}{2}\\ \frac{\sqrt{6}}{4} & -\frac{\sqrt{6}}{4} & 0\\ 0 & 0 & 1 \end{array}\right]\left[\begin{array}{c} G_{2}\\ G_{3}\\ G_{4} \end{array}\right]$$

In the case of combination of accelerometers 1, 3 and 4, the acceleration in different directions can be calculated as follows:

(19) $$\left[\begin{array}{c} G_{X2}\\ G_{Y2}\\ G_{Z2} \end{array}\right]=\left[\begin{array}{ccc} \frac{3\sqrt{2}}{4} & 0 & -\frac{\sqrt{2}}{4}\\ -\frac{\sqrt{6}}{4} & -\frac{2\sqrt{6}}{4} & \frac{\sqrt{6}}{4}\\ 0 & 0 & 1 \end{array}\right]\left[\begin{array}{c} G_{1}\\ G_{3}\\ G_{4} \end{array}\right]$$

In the case of combination of accelerometers 1, 2 and 4, the acceleration in different directions can be calculated as follows:

(20) $$\left[\begin{array}{c} G_{X3}\\ G_{Y3}\\ G_{Z3} \end{array}\right]=\left[\begin{array}{ccc} \frac{3\sqrt{2}}{4} & 0 & -\frac{\sqrt{2}}{4}\\ \frac{\sqrt{6}}{4} & \frac{\sqrt{6}}{2} & -\frac{\sqrt{6}}{4}\\ 0 & 0 & 1 \end{array}\right]\left[\begin{array}{c} G_{1}\\ G_{2}\\ G_{4} \end{array}\right]$$

In the case of combination of accelerometers 1, 2 and 3, different direction accelerations can be calculated as follows:

(21) $$\left[\begin{array}{c} G_{X4}\\ G_{Y4}\\ G_{Z4} \end{array}\right]=\left[\begin{array}{ccc} \frac{\sqrt{2}}{2} & -\frac{\sqrt{2}}{4} & -\frac{\sqrt{2}}{4}\\ 0 & \frac{\sqrt{6}}{4} & -\frac{\sqrt{6}}{4}\\ 1 & 1 & 1 \end{array}\right]\left[\begin{array}{c} G_{1}\\ G_{2}\\ G_{3} \end{array}\right]$$

For each data sample, the Earth’s gravitational acceleration in different combinations of sensors is calculated according to the following equations:

(22) $${\hat{G}}_{L2,3,4}\left(k\right)=\sqrt{G_{X1}^{2}\left(k\right)+G_{Y1}^{2}\left(k\right)+G_{Z1}^{2}\left(k\right)}$$

(23) $${\hat{G}}_{L1,3,4}\left(k\right)=\sqrt{G_{X2}^{2}\left(k\right)+G_{Y2}^{2}\left(k\right)+G_{Z2}^{2}\left(k\right)}$$

(24) $${\hat{G}}_{L1,2,4}\left(k\right)=\sqrt{G_{X3}^{2}\left(k\right)+G_{Y3}^{2}\left(k\right)+G_{Z3}^{2}\left(k\right)}$$

(25) $${\hat{G}}_{L1,2,3}\left(k\right)=\sqrt{G_{X4}^{2}\left(k\right)+G_{Y4}^{2}\left(k\right)+G_{Z4}^{2}\left(k\right)}$$

For sample *N*, the MAE of acceleration calculations for three sensor combinations can be obtained using the following equations:

(26) $${\mathrm{MAE}}_{2,3,4}=\frac{\sum_{k=1}^{N}\left|{\hat{G}}_{L2,3,4}\left(k\right)-G_{L}\right|}{N}$$

(27) $${\mathrm{MAE}}_{1,3,4}=\frac{\sum_{i=1}^{N}\left|{\hat{G}}_{L1,3,4}\left(k\right)-G_{L}\right|}{N}$$

(28) $${\mathrm{MAE}}_{1,2,4}=\frac{\sum_{i=1}^{N}\left|{\hat{G}}_{L1,2,4}\left(k\right)-G_{L}\right|}{N}$$

(29) $${\mathrm{MAE}}_{1,2,3}=\frac{\sum_{i=1}^{N}\left|{\hat{G}}_{L1,2,3}\left(k\right)-G_{L}\right|}{N}$$

If only one of the accelerometers is faulty, the mean of calculated errors will not be within an acceptable error limit. This means that the calculated average by other sensors meets the condition and the defective sensor is detected. In this case, if more than one sensor is defective, none of the scenarios will satisfy the condition. It means that more than one sensor in the four sensor system is defective.

In the mode of using n sensors (n>3) and in the case of fault on m sensors, the proposed method can generally detect these m sensors if n−m≥3. This means that by increasing the number of sensors, faultier sensors can be detected. In the case of excessive redundancy of sensors, the number of calculations is increasing. If the number of sensors from n sensors increases to n+1 sensors, then the number of possible triple combinations and calculations will increase up to $\frac{n+1}{n-2}$ times. For example, if four sensors are used, the number of possible combinations is four cases. If five sensors are used, the number of possible combinations is 10 cases. As a result, the calculation will increase 2.5-fold.

The following algorithm is proposed for auto-calibration and fault diagnosis and isolation of SRAMWD with optimal skewed structure. The algorithm has been tested using real data in this study. 

Initial auto-calibration of redundant accelerometers with skewed structure considering of all sensors and determination of calibration coefficients including biases, scale factors, and misalignment of sensors:

1. Initial auto-calibration of the combinations of three sensors from four redundant accelerometers, assuming the use of three sensors in each case and determining the calibration coefficients in each case.
2. Calculation of the Earth’s gravitational acceleration using the redundant accelerometers of skewed structure on each sample of data.
3. If there is an overlimit difference between the calculated gravitational acceleration and the actual gravitational acceleration, sample more data.
4. Calculate the mean error in the gravitational acceleration calculation in these samples of data.
5. Fault diagnosis by checking acceptable limit for error is defined in Equation (15).
6. In fault diagnosis, calculate the MAE of gravitational acceleration with different combinations of three sensors, assuming the use of three accelerometers out of four available ones and considering the calibration coefficients for each case, are defined in Equations (26)–(29).
7. Detect the faulty accelerometer by checking the limit condition of the difference between the MAE of accelerations and the real amount of gravitational acceleration.
8. Isolate and remove the faulty accelerometer from the calculation.
9. Checking the MAE condition limit of the calculated gravitational acceleration error using the remaining correct accelerometers.
10. If necessary, perform a new auto-calibration based on the remaining correct accelerometers and calculate the calibration coefficients of the correct accelerometers.
11. Continue the process with the correct accelerometers.

## 5. Experimental Results

**Figure 2:**
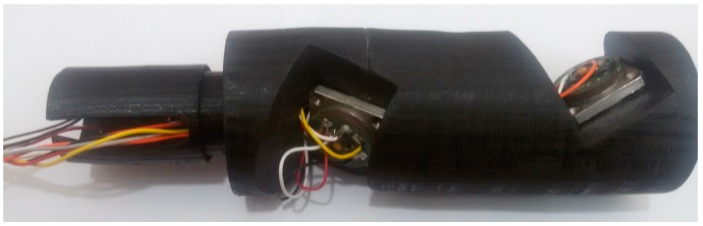
The tool with skewed structure including four redundant accelerometers.

**Figure 3:**
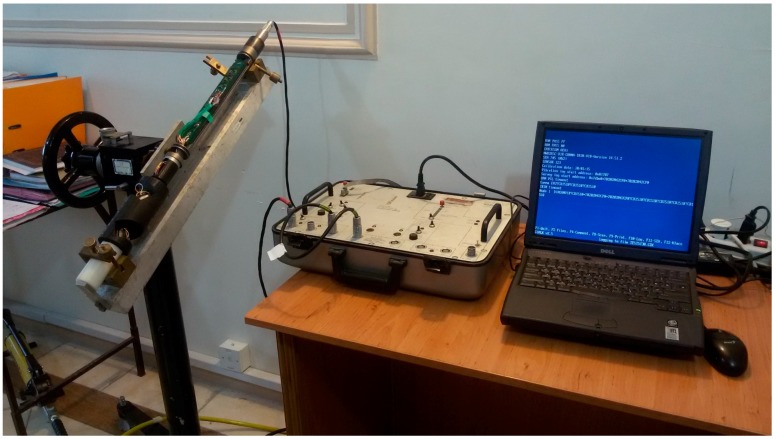
The tool kit with 4 redundant accelerometers, clamp, drill simulator, and monitoring system.

To implement the proposed system, the optimal skewed 4-sensor structure was initially selected according to the structure of four redundant accelerometers, given in Figure 1. The mechanical design proportional to the shape of the device for the implementation of the current MWD tool is performed. The selected optimal design structure is then built and the accelerometers are installed. This process is shown in Figure 2. The test set suit with four redundant accelerometers, clamp, drill simulator, and monitoring system is illustrated in Figure 3. 

### 5.1. Auto-Calibration of SRAMWD

The auto-calibration is performed on new SRAMWD instrument in the case of four sensors and the biases, the scale factors, and the misalignments of accelerometers are estimated. In addition, auto-calibration of the three-sensor cases is executed. To perform these operations in different circumstances, the tool will turn around itself and the data will be sampled. The initial values of the auto-calibration coefficients in the optimization algorithm are considered as the value provided by the manufacturer. 

**Figure 4:**
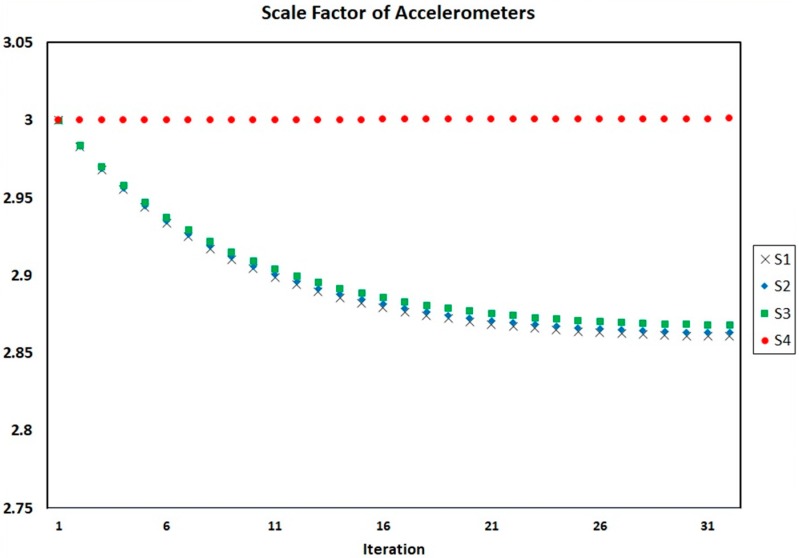
Biases of four accelerometers in each iteration of auto-calibration.

**Figure 5:**
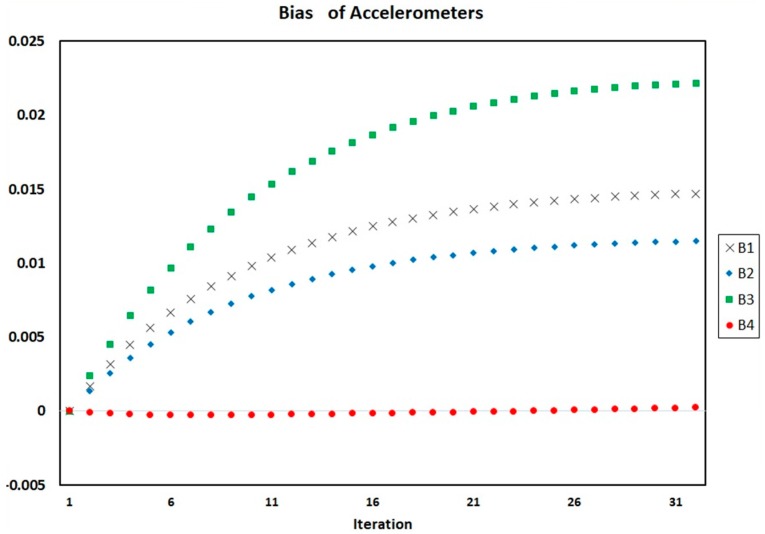
Scale factors of four accelerometers in each iteration of auto-calibration.

**Figure 6:**
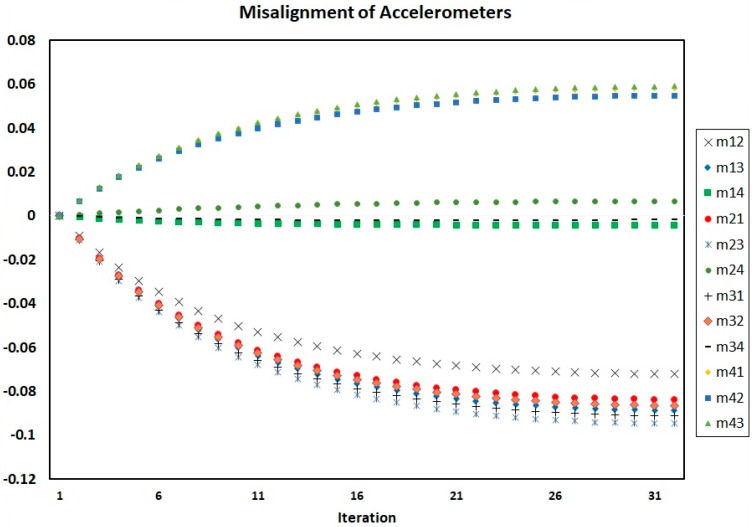
Misalignments of four accelerometers in each iteration of auto-calibration.

Figure 4, Figure 5 and Figure 6 show the process of calculation and convergence of bias parameters, scale factors, and misalignments of four accelerometers, respectively. Similarly, the figures clearly indicate that the auto-calibration operation of four skewed redundant accelerometers has been performed well via nonlinear optimization procedure and the unknown parameters containing biases, scale factors, and misalignments after repeated several times are converged and estimated optimally.

**Figure 7:**
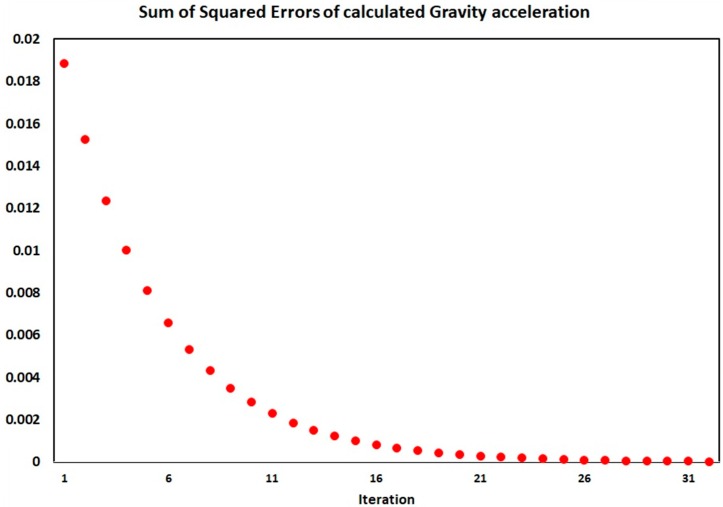
Sum of squared errors of gravity acceleration in each iteration of SRAMWD auto-calibration.

Figure 7 shows the sum of squared errors of gravitational acceleration calculation for each iteration. As shown in the figure, after the repetition of auto-calibration operation, the sum of squared errors of the gravitational acceleration is almost zero and this shows the optimal measurement of the accelerometers parameters.

**Figure 8:**
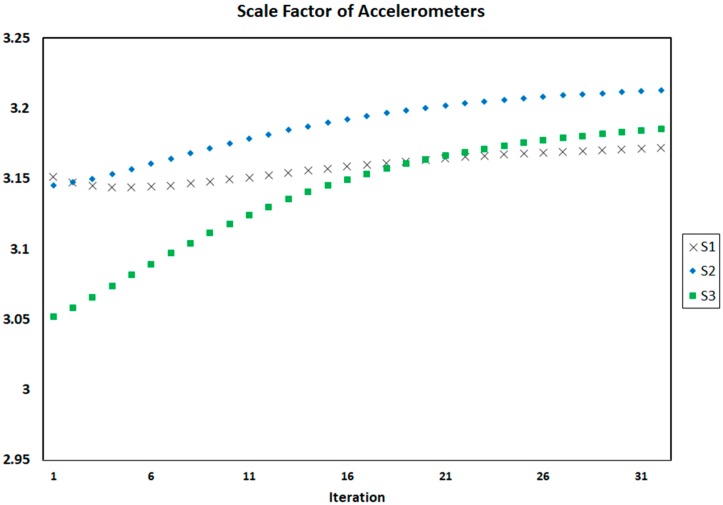
Biases of accelerometers on each iteration of auto-calibration of SRAMWD tool for combination status of accelerometers number 1, 2 and 3.

**Figure 9:**
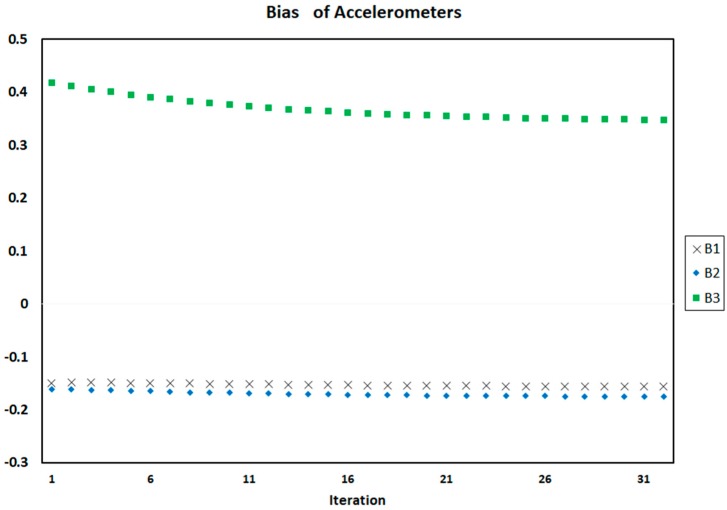
Scale factors of accelerometers on each iteration of auto-calibration of SRAMWD tool for combination status of accelerometers number 1, 2 and 3.

**Figure 10:**
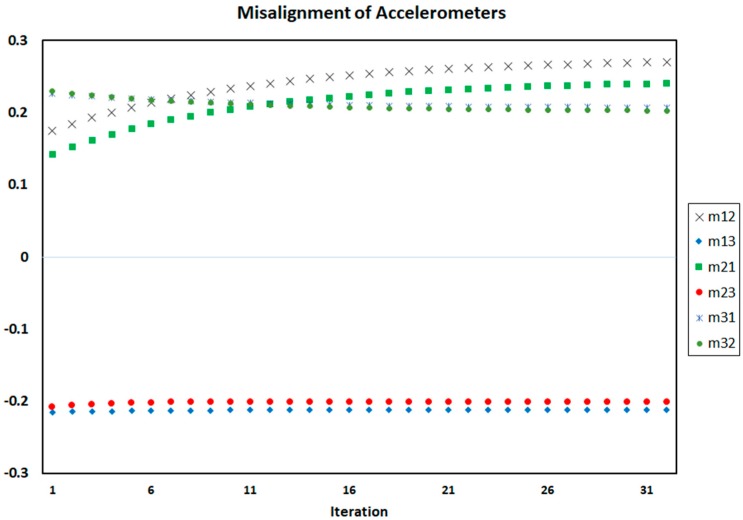
Misalignments of accelerometers on each iteration of auto-calibration of SRAMWD tool for combination status of accelerometers number 1, 2 and 3.

According to the FDI algorithm, it is necessary to calculate the calibration coefficients of the 3-sensor cases. Therefore, auto-calibration is performed for all 3-sensor cases. Figure 8, Figure 9 and Figure 10 show the bias parameters, scale factors, and misalignments for each auto-calibration iteration using three accelerometers numbered 1, 2 and 3, respectively. For other cases using three accelerometers, similar results were obtained.

**Figure 11:**
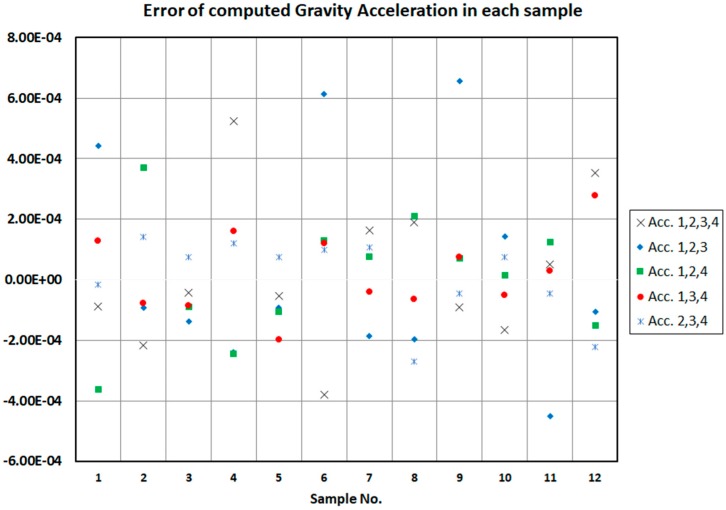
Gravitational acceleration error through all four sensors and each combination status of three accelerometers.

In the end, the sampling was performed based on the new structure of the suggested model. To test the performance of the model, sample data categories at various positions were collected. Figure 11 shows the gravitational acceleration errors calculated through all four sensors and different combinations of three sensors. Similarly, it can be seen, the error of calculating the gravitational acceleration when using all four accelerometers and different combination of three accelerometers is very low. According to these figures, it can be seen that the auto-calibration is accurately implemented.

### 5.2. FDI in SRAMWD

By examining the operation on the faulty accelerometers on the MWD tools under repair, it was found that when a fault occurs in an accelerometer, the accelerometer voltage would have different values such as 0 V, 3 V, −3 V or a random amount between these values. Therefore, in the sensor data for each faulty accelerometer, the value of 0, −3, 3 or random amount between −3 to 3 is considered. At that moment, the FDI is implemented in accordance with the proposed algorithm. The real amount of gravitational acceleration based on Equations (8) and (9) and according to the location of the experiments is considered 0.9995 G. The error limit is considered to be 0.05 G for drilling operational tests.

The results of the experiments are shown in Table 2. According to the results of the experiment number 1, there is no faulty sensor and the accelerometers are correct. It appears that the algorithm does not reveal the existence of a faulty accelerometer. In experiments number 2 to number 17, a faulty sensor was considered, and different sensor voltage values were considered for the faulty accelerometer.

First, the proposed FDI procedure detected that there is a faulty accelerometer, then the faulty sensor is isolated. It is clear that the MAE in cases of using faulty accelerometer is noticeably different from the MAE in case of using correct accelerometers. When the faulty sensor is used, the MAE is clearly increased. In the experiments number 18 to number 23, two faulty sensors are placed on the tool and tested in different cases. It is shown that in these experiments, MAE is clearly higher than the limit condition. Therefore, in this regard, the existence of fault is detected but the faulty sensors are not detectable.

## 6. Conclusions

This study adopted a four-sensor structure of optimal structure proposed by Shim et al. [8]. The appropriate design using an existing tool has been achieved. The new SRAMWD structure was built and installed on the current system. The auto-calibration of the new SRAMWD was successfully implemented using the Newton-Raphson Nonlinear Optimization Method. In addition, by causing intentional faults on the sensors on the new structure, the faults were revealed using the proposed FDI method based on data and selection of the appropriate feature. Therefore, the faulty sensor was diagnosed and isolated.

## Figures and Tables

**Table 1:** Parameter definition.

Description	Parameter
Ambient temperature, measured by the thermometer	$T_{s}$
The nominal temperature, which is typically 25 °C	$T_{C}$
The scale factor independent of the temperature of the *i*-th sensor	$SF_{i}$
The scale factor depended on the first-degree temperature of the *i*-th sensor	$SFT1_{i}$
The scale factor depended on the second-degree temperature of the *i*-th sensor	$SFT2_{i}$
The scale factor depended on the third-degree temperature of the *i*-th sensor	$SFT3_{i}$
The bias independent of the temperature of the *i*-th sensor	$B_{i}$
The bias dependent on the first-degree temperature of the *i*-th sensor	$BT1_{i}$
The bias dependent on the second-degree temperature of the *i*-th sensor	$BT2_{i}$
The bias dependentt on the third-degree temperature of the *i*-th sensor	$BT3_{i}$
Measured voltage of *i*-th accelerometer	$V_{i}$
Acceleration in the direction of the *i*-th accelerometer	$G_{i}$
Misalignment Parameter between sensor *i* and sensor *j* $m_{ii}=0$	$m_{ij}$

**Table 2:** Results of FDI algorithm in different cases (Acc.: Accelerometer, MAE: Mean Absolute Error).

Test No.	Faulty Acc.	Voltage of Faulty Acc.	MAE Using 4 Acc.	Fault Detection	MAE for 3 Acc. 1, 2, 3	MAE for 3 Acc. 1, 2, 4	MAE for 3 Acc. 1, 3, 4	MAE for 3 Acc. 2, 3, 4	Isolated Acc.
1	-	-	0.000212	No	0.000705	0.000763	0.000512	0.000758	-
2	1	0	0.1454	Yes	0.2063	0.1456	0.1312	0.000758	1
3	1	−3	0.2073	Yes	0.2830	1.0463	0.9643	0.000758	1
4	1	+3	0.1462	Yes	0.3541	0.2404	0.2503	0.000758	1
5	1	Rand	0.1225	Yes	0.2080	0.2837	0.2285	0.000758	1
6	2	0	0.1369	Yes	0.2131	0.1537	0.000512	0.1417	2
7	2	−3	0.2042	Yes	0.2703	1.0223	0.000512	0.9959	2
8	2	+3	0.1522	Yes	0.3517	0.2534	0.000512	0.2743	2
9	2	Rand	0.1282	Yes	0.2645	0.2073	0.000512	0.1918	2
10	3	0	0.1523	Yes	0.1721	0.000763	0.1469	0.1471	3
11	3	−3	0.1950	Yes	0.8378	0.000763	0.9747	1.0218	3
12	3	+3	0.1472	Yes	0.4670	0.000763	0.2477	0.2560	3
13	3	Rand	0.1264	Yes	0.2343	0.000763	0.1927	0.2304	3
14	4	0	0.3107	Yes	0.000705	0.3669	0.3390	0.3323	4
15	4	−3	0.1736	Yes	0.000705	0.8509	0.9739	0.8332	4
16	4	+3	0.1850	Yes	0.000705	0.2332	0.2636	0.2347	4
17	4	Rand	0.1979	Yes	0.000705	0.2722	0.4745	0.4751	4
18	1 and 2	Rand	0.2310	Yes	0.3291	0.7170	0.3398	0.2550	-
19	1 and 3	Rand	0.2381	Yes	0.3591	0.2520	0.5747	0.3349	-
20	1 and 4	Rand	0.2296	Yes	0.2496	0.4378	0.4715	0.3351	-
21	2 and 3	Rand	0.2148	Yes	0.3052	0.4401	0.2454	0.3970	-
22	2 and 4	Rand	0.2268	Yes	0.2169	0.5142	0.4373	0.3788	-
23	3 and 4	Rand	0.2457	Yes	0.3235	0.4539	0.2616	0.2775	-
